# Left ventricular non-compaction cardiomyopathy and left ventricular assist device: a word of caution

**DOI:** 10.1186/s13019-016-0503-2

**Published:** 2016-07-15

**Authors:** A. Kornberger, U. A. Stock, P. Risteski, A. Beiras Fernandez

**Affiliations:** Department of Thoracic and Cardiovascular Surgery, University Hospital Goethe University, Theodor-Stern-Kai 7, 60590 Frankfurt am Main, Germany

**Keywords:** Left ventricular non-compaction, Left ventricular assist device, Inflow obstruction, Heart transplantation

## Abstract

**Background:**

In patients with left ventricular non-compaction (LVNC), implantation of a left ventricular assist device (LVAD) may be performed as a bridge to transplantation. In this respect, the particular characteristics of the left ventricular myocardium may represent a challenge.

**Case presentation:**

We report a patient with LVNC who required urgent heart transplantation for inflow cannula obstruction nine months after receiving a LVAD. LVAD parameters, echocardiography and examination of the explanted heart suggested changes of left ventricular configuration brought about by LVAD support as the most likely cause of inflow cannula obstruction.

**Conclusions:**

We conclude that changes experienced by non-compacted myocardium during LVAD support may give rise to inflow cannula obstruction and flow reduction. Presence of LVNC mandates tight surveillance for changes in LV configuration and LVAD flow characteristics and may justify urgent transplantation listing status.

## Background

Left ventricular non-compaction (LVNC) is attributed to arrest of normal embryogenesis of the endocardium and myocardium and results in a two-layered structure of the left ventricle (LV). In addition to a compacted outer layer, the LV comprises a spongy non-compacted inner layer with heavy trabeculation and deep intertrabecular recesses [1, 2]. Though its incidence and prevalence are still uncertain, LVNC was recently suggested to represent the third most commonly diagnosed cardiomyopathy [3]. The major clinical findings in LVNC are heart failure, arrhythmias and thromboembolic events [1, 2]. In patients with end-stage heart failure, LVAD implantation may be performed as a bridge to transplantation [4–7].

While previous authors focused on thromboembolic events [5, 7] and arrhythmia [7] associated with LVAD support in LVNC, the changes non-compacted left ventricular myocardium may experience during LVAD support also merit investigation. Our case sheds light on LVAD support in LVNC from a different angle and raises questions with regard to the duration of support being likely to be limited by support-induced changes of non-compacted myocardium, LV configuration and LVAD flow behaviour.

## Case report

A 35-year-old male with LVNC underwent off-pump LVAD implantation (HVAD, HeartWare Inc.) through a minimally invasive access. Presence of trabeculation obstructing the inflow cannula was excluded by digital exploration before introduction of the LVAD. Correct inflow cannula position and absence of obstruction were additionally verified by transesophageal echocardiography and evaluation of LVAD flow behaviour before closure. After an uneventful hospital stay and subsequent exercise tolerance and quality of life improvement, the patient presented with low flow alarms and had his LVAD speed re-adjusted several times. After nine months, he was readmitted for perilously low flows that intermittently dropped to 500 ml/min. This was interpreted as the consequence of an excess of outdoor activity and volume depletion, and when LVAD flows increased satisfactorily after administration of fluids, the patient was discharged the next day. Subsequently, LVAD flows kept dropping intermittently over a few weeks, and finally the patient ceased to respond to administration of fluids and developed signs of heart failure. He was re-admitted, and his waiting list status was set to high urgency.

**Fig. 1:**
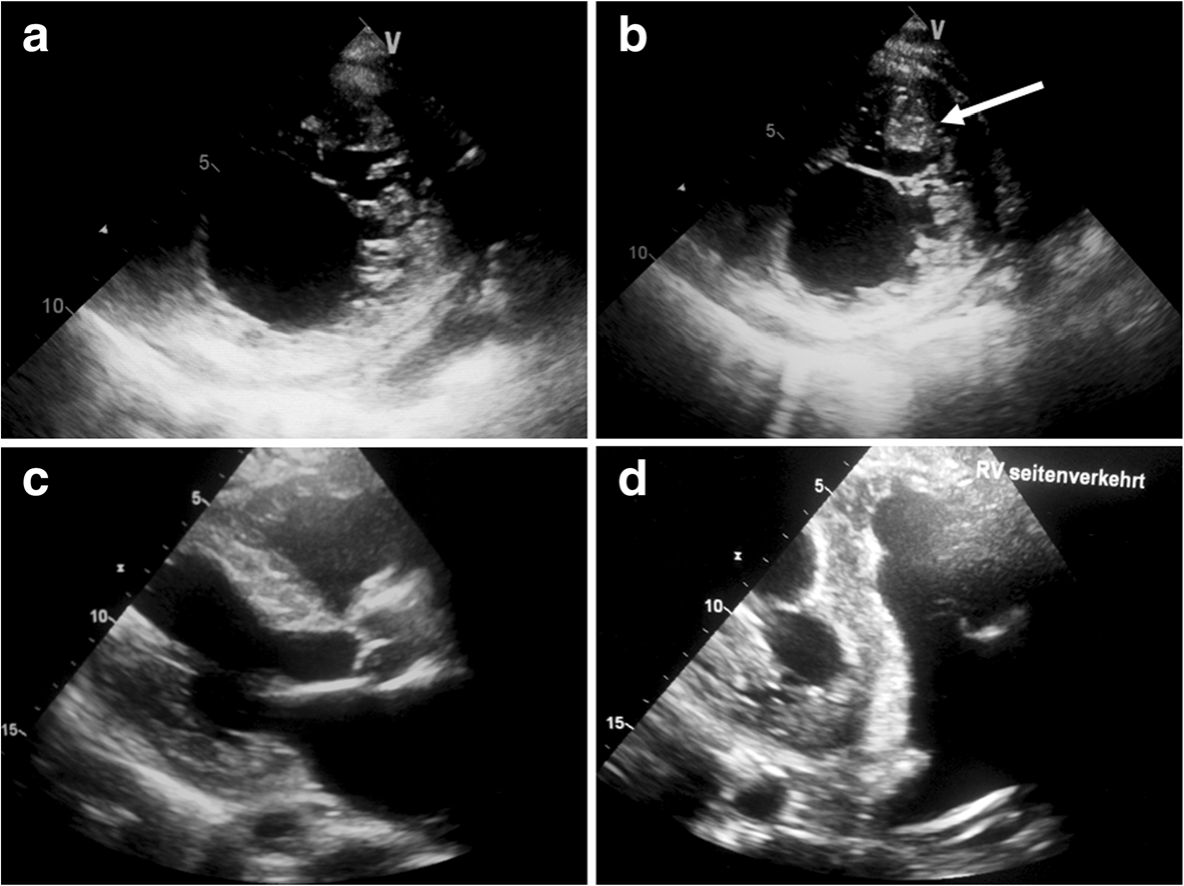
Transthoracic echocardiography 9 months after LVAD implantation: **a** short axis view showing trabeculated left ventricular myocardium; **b** tilted short axis view showing a solid structure stretching across the LV in front of the LVAD inflow cannula (arrow), **c** parasternal long axis view showing thick, spongy LV myocardium and narrow LV cavity, **d** inverted tilted four chamber view showing thickened LV myocardium with small LV cavity appearing divided by a solid structure

After four weeks of medical treatment, he was put on extracorporeal life support (ECLS). By that time, changes in LV configuration had come to be suspected as the cause of LVAD dysfunction. Echocardiography showed the LV cavity smaller and the LV walls thicker than before LVAD implantation, with heavy trabeculation within the LV and a solid structure appearing to cross in front of the inflow cannula opening (Fig. 1).

**Fig. 2:**
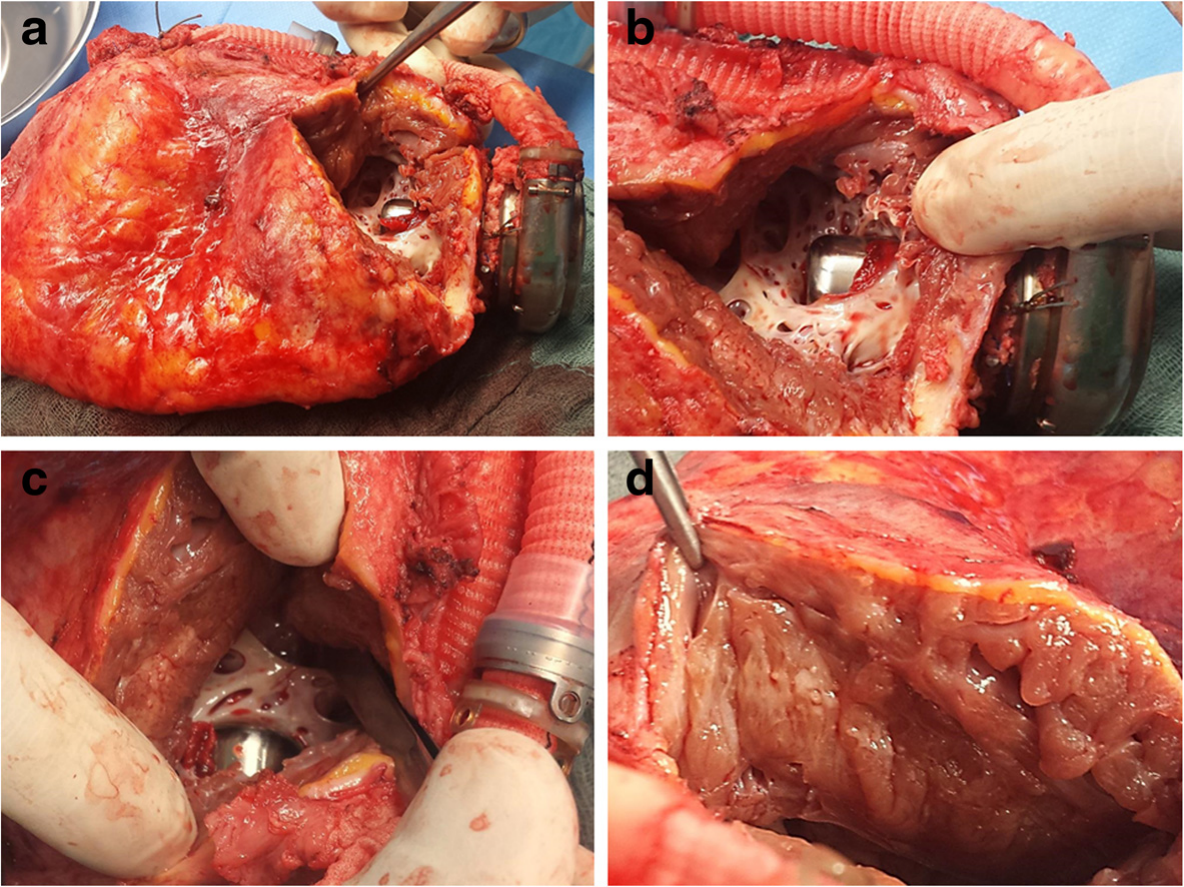
Heart explanted after nine months of LVAD support: **a** HVAD inflow cannula protruding into massively enlarged heart, **b** trabeculated tissue surrounding the inflow cannula, **c** dense stretch of trabeculated tissue obstructing the opening of the inflow cannula within partially obliterated apex, **d** thick, spongy myocardium interspersed with fatty deposits

After 45 days on ECLS, the patient underwent heart transplantation. Macroscopic examination of the explanted heart confirmed the findings on echocardiography. The LV cavity was narrow and partially obliterated by dense tissue. In particular, there was a rigid trabecular structure stretching across the LV in front of and appearing to have overgrown the LVAD inflow cannula. The left ventricular wall showed massive thickening to nearly 30 mm, with the myocardium spongy and interspersed with yellowish fatty deposits (Fig. 2).

## Discussion

LVNC was found to lead to severe heart failure in a considerable share of those diagnosed with the condition, with 47 % [1] and 60 % [8] reported to have either died or undergone transplantation within a mean follow-up period of 44 ± 40 months and within 6 years of diagnosis, respectively. Nevertheless, reports on VAD implantation in patients with LVNC are infrequent, especially when those featuring pediatric patients supported with pulsatile systems [9–11] are left aside (Table 1).

**Table 1:** Outcomes of LVAD implantation in patients with left ventricular non-compaction

	No.	Patients	Device	Duration of support	Complications	Outcome
Maile et al., 2004 [4]	1	Male, 51 years	DeBakey MicroMed LVAD	2.5 months	None	Successfully bridged to transplantation
Ivan et al., 2005 [6]	2	Male, 21 years	Jarvik biventricular assist device	Not indicated	Not indicated	Death before transplantation
		Male, 37 years	Thoratec RVAD and HeartMate LVAD	Not indicated	Not indicated	Death after transplantation
Nathan et al., 2010 [9]	1	Male, 14 years	Abiomed, biventricular assist device	5 days	None	Successfully bridged to transplantation
Ninios et al., 2010 [5]	1	Male, 28 years	Jarvik 2000 LV	3 years	Recurrent thrombosis with successful intravenous lysis	Patient on LVAD support at the date of publication
Hanke et al., 2012 [10]	1	Male, 12 months	Berlin Heart, Excor, biventricular assist device	24 days	Pulmonary haemorrhage requiring use of a membrane oxygenator, thrombosis requiring replacement of the LVAD	Successfully bridged to transplantation
Siehr et al., 2013 [11]	1	Male, 6 weeks	Berlin Heart, Excor, LVAD	56 days	Embolic cerebrovascular accident, 2 pump exchanges due to clot formation	Successfully bridged to transplantation
Uribarri et al., 2015 [7]	5	4 males (23, 25, 43,54 years) 1 female (24 years)	3 HVAD 2 HeartMate II	Mean follow up period 86.5 weeks, range 40–194 weeks	4 cases of pump thrombosis in 3 of 5 patients	2 deaths, 3 patients on LVAD support at the date of publication

Ninios et al. [5] reported off-pump implantation of a Jarvik 2000 LV via a left posterolateral thoracotomy, while Maile et al. [4] described a case of implantation of a DeBakey LVAD in a patient with LVNC. 5 patients reported by Uribarri et al. [7] underwent on-pump HeartMate II implantation through a full sternotomy or on-pump HVAD implantation through an upper hemisternotomy and left-sided anterolateral thoracotomy. In this series, thrombosis was the main issue, with arrhythmia investigated as a second outcome parameter [7]. In the case reported by Ninios et al., the focus was also on the association between LVNC and pump thrombosis [5]. The patient reported by Maile et al. [4] underwent transplantation after 2.5 months of uneventful LVAD support, and Ivan et al. [6] reported two cases that took a fatal course after biventricular assist devices were implanted as a last remedy. In none of the cases published to date, however, mention was made of complications resulting from the particular structure of the left ventricular myocardium in patients with LVNC even though some of the patients were on LVAD support for considerably longer periods than our patient [5, 7] and Uribarri et al. [7] explicitly reported the apex to be the region that was most frequently affected by non-compaction in their patients.

The latter finding is in keeping with previous literature reporting the typical features of LVNC to be located predominantly in the apical and mid-ventricular segments of the LV [1], which is relevant in that trabecular structures within a nearly obliterated apex may result in obstruction of the inflow cannula. In this respect, the implantation method may be of relevance, because on-pump implantation allows more thorough inspection and palpation of the left ventricle in order to exclude presence of or remove apical trabecular structures that may result in obstruction of the inflow cannula.

In our case, off-pump HVAD implantation was uncomplicated, obstruction of the inflow cannula by apical trabecular structures was excluded by digital palpation as well as by transesophageal echocardiography, and unhindered blood flow into the inflow cannula was reflected by normal LVAD flow characteristics. In hindsight, however, on-pump implantation would have allowed not only inspection of the left ventricular apex, but also excision of excess tissue. On the other hand, the fact that first signs of inflow cannula obstruction occurred after several months of uneventful support and absence, in earlier echocardiographic examinations, of the heavy apical and mid-ventricular trabecular masses seen on echocardiography after readmission for LVAD dysfunction suggest that at least part of the changes that finally resulted in inflow cannula obstruction must have occurred over time.

While decompression of the LV is a normal and desirable consequence of LV unloading, the extreme degree of narrowing of the LV cavity we saw in the explanted heart as well as the heavy mass of rigid trabeculated tissue around the inflow cannula represent unusual findings. They are most likely explained by LV configuration and the geometry and density of pre-existing trabeculated tissue having changed in response to the hemodynamic changes that were brought about by LVAD support.

## Conclusion

We conclude that LVNC may turn out to be a cause of inflow cannula obstruction and flow reduction even where LVAD function was normal upon implantation. On-pump implantation may be advisable with a view to inspecting the inflow opening and excising excess tissue, thus preventing subsequent catastrophic inflow obstruction. LVNC requires a high index of suspicion and mandates tight surveillance of LVAD flow characteristics and LV configuration. If LVNC actually turns out to be a limiting factor in LVAD therapy, it may justify more liberal access to high urgency listing for patients afflicted with this particular condition.

## Abbreviations

ECLS, extracorporeal life support; HVAD, left ventricular assist device supplied by HeartWare Inc; LV, left ventricle, left ventricular; LVAD, left ventricular assist device; LVNC, left ventricular non compaction; VAD, ventricular assist device
